# An Anterolateral Popliteal Artery Puncture Resulting in an Arteriovenous Fistula Involving Three Vessels: Bail Out by Balloon Hemostasis

**DOI:** 10.7759/cureus.83474

**Published:** 2025-05-04

**Authors:** Daisuke Yamazaki

**Affiliations:** 1 Cardiology, Akita Cerebrospinal and Cardiovascular Center, Akita, JPN

**Keywords:** anterolateral popliteal artery puncture, arteriovenous (av) fistula, distal artery approach, endovascular therapy (evt), peripheral arterial diseases

## Abstract

The anterolateral popliteal artery (PA) puncture technique is mainly used during endovascular therapy of the superficial femoral artery when antegrade wiring fails to pass the wire through the true lumen of the distal artery. This method has many advantages, such as allowing puncture while the patient is in the supine position and relatively easy hemostasis; however, the distance from the body surface to the PA is long, and puncture requires practice. In this case, the distal artery approach was performed for the treatment of chronic occlusive lesions in the left superficial femoral artery. We performed anterolateral PA puncture, inserted a 4.0-Fr sheath, and successfully achieved revascularization using a controlled antegrade and retrograde tracking (CART) technique. Post-procedural angiography after sheath removal revealed an arteriovenous fistula between the fibular artery and fibular vein and another arteriovenous fistula between the fibular artery and the anterior tibial vein. After adding two 10-minute balloon hemostasis, the arteriovenous shunt almost disappeared, and there was no recurrence of the arteriovenous shunt thereafter. The patient has been an outpatient for four years with no recurrence of arteriovenous shunts. Repeated punctures due to inexperience with anterolateral PA puncture and the insertion of a sheath at a low peripheral site may increase the risk of arteriovenous fistula. Becoming skilled at puncture and puncturing the PA at the height of the fibular head can reduce the risk of arteriovenous fistula.

## Introduction

The anterolateral popliteal artery (PA) puncture is a puncture technique reported in 2017. It is mainly used in endovascular therapy (EVT) for occluded lesions of the superficial femoral artery (SFA) when the guidewire cannot pass through the lesion by the antegrade approach alone, and a distal artery approach is required [1]. Compared with the posterior PA puncture, which is performed in the prone position [2], and the foot raised PA puncture, which requires the lower limb to be elevated [3], the anterolateral PA puncture can be performed in the supine position, making its preparation easier. Furthermore, it is easier to achieve hemostasis than SFA puncture distal to the adductor canal [4]. Compared with below-the-knee artery puncture, it often remains an approach site. Thus, anterolateral PA puncture is a technique with many advantages. However, some familiarity is required because the distance from the body surface to the PA is long, approximately 4.0-5.0 cm [1,5], and the puncture must pass between the tibia and fibula. Due to the distance from the body surface to the PA and the presence of the tibia and fibula in front, puncture under echo guidance is also difficult. There are still a few original articles on anterolateral PA puncture, but there are reports suggesting anatomical landmarks to simplify the procedure [5,6].

In this case, EVT was performed for an occluded lesion in the left SFA, but the wire could not pass through the true peripheral lumen using only the conventional knuckle wire technique. An arteriovenous fistula was formed in the anterior tibial vein, fibular vein, and tibial artery during the anterolateral PA puncture, and the patient was bailed out by balloon hemostasis.

## Case presentation

An 80-year-old woman was admitted to our institution with a chief complaint of diplopia and lightheadedness. A head MRI revealed a cerebral infarction in the left thalamus. She was treated conservatively, underwent rehabilitation, and complained of intermittent claudication of the left lower limb. The blood pressure pulse wave revealed an ankle brachial pressure index (ABI) of 0.91/0.45. A lower limb artery ultrasound revealed a left SFA occlusion of approximately 3 cm. The patient had peripheral arterial disease (PAD) with intermittent claudication and was unable to perform adequate exercise therapy due to the sequelae of cerebral infarction; therefore, the decision was made to perform EVT.

**Figure 1:**
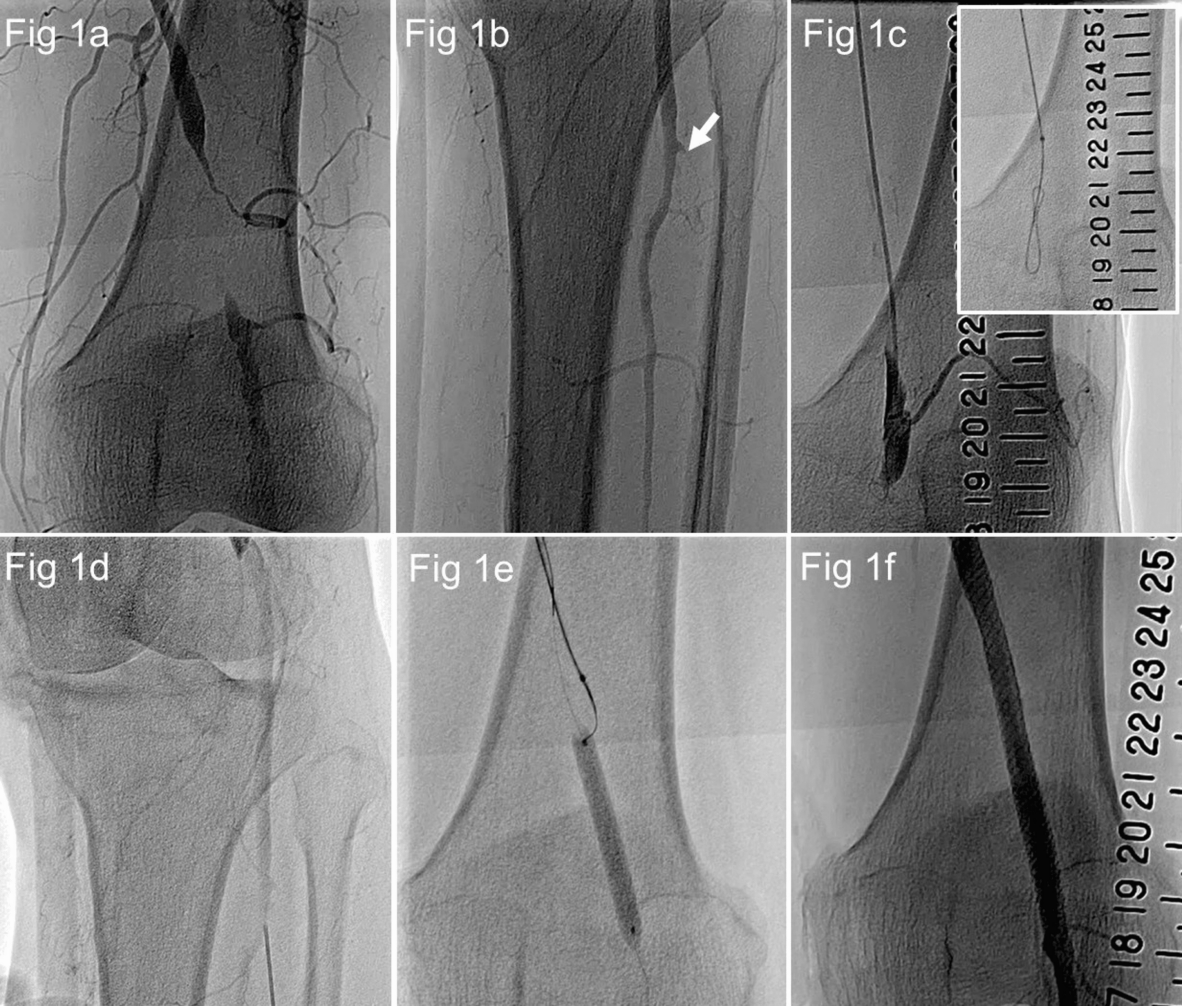
Process of endovascular therapy. (1a) Lower extremity arteriography. The left superficial femoral artery was occluded for approximately 3 cm. (1b) Regarding the below-knee artery, only the peroneal artery showed good patency. The anterior tibial artery was occluded at the entry. (1c) A 0.035-inch guidewire was used for the knuckle wiring. However, it did not pass through the true lumen of the peripheral artery. (1d) Anterolateral PA puncture was performed. The puncture vessel was later identified as the fibular artery. (1e) A 0.014-inch guidewire was passed into the true lumen of the peripheral artery using the controlled antegrade and retrograde tracking (CART) technique. (1f) Endovascular therapy was successfully performed by implanting a SuperaⓇ stent after balloon dilatation.

**Figure 2:**
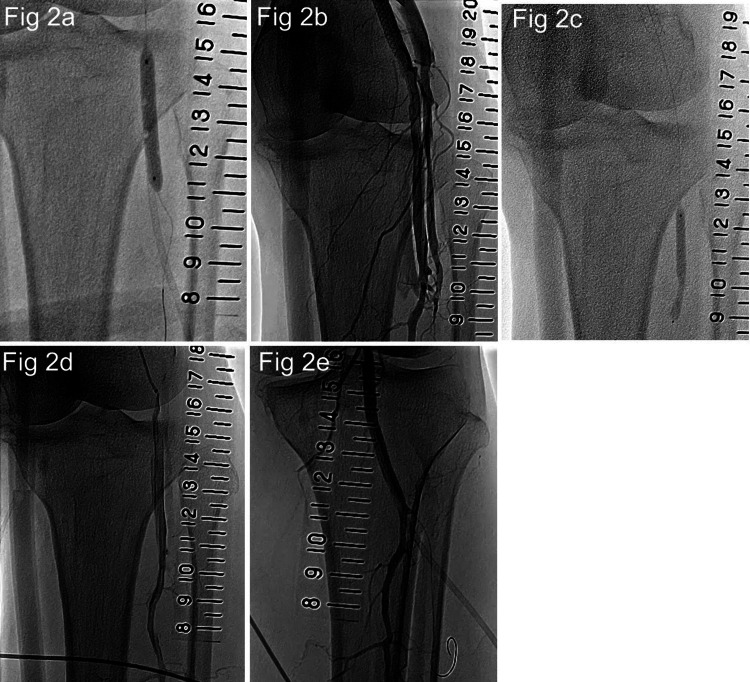
The process from sheath removal to bailing out the arteriovenous fistula. (2a) A 4.0-Fr sheath was removed during balloon hemostasis. (2b) Arteriovenous fistulas were observed between the fibular artery and popliteal vein and between the fibular artery and anterior tibial vein. (2c) Balloon dilatation was performed for 10 minutes at the site of the arteriovenous fistula, and this procedure was repeated twice. (2d) The shunt flow to the fibular vein disappeared after the first additional dilation, and the shunt flow to the anterior tibial vein also almost disappeared after the second additional dilation. (2e) Lower limb angiography with a sheath inserted. The guidewire is advanced into the anterior tibial artery, and the sheath is inserted into the fibular artery.

EVT was performed for PAD with intermittent claudication. The right femoral artery was punctured, and a 6.0-Fr guiding sheath (Parent Plus60 55 cm; Medikit, Tokyo, Japan) was inserted. Lower limb angiography revealed a 3-cm occlusive lesion distal to the SFA (Figure 1a). The peripheral portion was contrasted by collateral vessels, and the anterior and posterior tibial arteries were occluded in the below-knee artery (Figure 1b). The occlusion was wired with a knuckle wire using ELITECROSSⓇ (Cordis, Tokyo, Japan) and Radifocus GuidewireⓇ 0.035-inch, J-curve 220 cm (Terumo, Tokyo, Japan). However, the peripheral true lumen was not accessed, so the distal artery approach was performed (Figure 1c). The anterior and posterior tibial arteries were occluded and could not be chosen as access sites, so we decided to approach from the PA using the anterolateral PA puncture technique (Figure 1d). This was the first anterolateral PA puncture performed at our institution. It took some time to puncture, but we could insert the IntroducerⓇ 4.0 Fr sheath (Terumo, Tokyo, Japan). The distal SFA was dilated by SABERXⓇ 5.0 × 40 mm from the PA, and a ChevalierⓇ 0.014-inch 30 g guide wire (NIPRO, Osaka, Japan) was used for the controlled antegrade and retrograde tracking (CART) technique [7]. The guide wire was reentered into the distal true lumen (Figure 1e). Subsequently, after balloon dilation, SuperaⓇ stent 6.0 × 60 mm (Abbott, Lake Bluff, IL) was implanted (Figure 1f). The sheath was removed while dilating for 10 minutes with ULTRAVERSE RXⓇ 4.0 × 40 mm (Becton Drive, Franklin Lakes, NJ) at the site where the sheath was inserted by anterolateral PA puncture (Figure 2a). The EVT process is also shown in Video 1.

**Video 1:** Process of endovascular therapy. A process of revascularization with the antero-lateral popliteal artery puncture and controlled antegrade and retrograde tracking (CART) technique.

Contrast was performed to confirm hemostasis, which showed the formation of arteriovenous fistulas traversing from the sheath insertion site to the bilateral veins (Figure 2b). Two additional cycles of 10-minute balloon hemostasis were performed (Figure 2c). The next day, a lower limb ultrasound revealed that the arteriovenous shunt had disappeared. Since then, the patient has been an outpatient for four years with no recurrence of arteriovenous shunts. Outpatient follow-up did not include regular ultrasound scans, but physical findings such as shunt noises and edema were assessed. The process of shunt blood flow loss with repeated balloon hemostasis is shown in Video 2.

**Video 2:** Process from sheath removal to bailing out the arteriovenous fistula. The process of shunt blood flow loss with repeated balloon hemostasis is shown in Video 2.

## Discussion

In this case, the antegrade guidewire did not pass the peripheral true lumen of the occluded SFA lesion. Thus, the distal artery approach was added, and the patient was successfully revascularized using the CART technique [7]. When performing the distal artery approach, the dorsal or posterior tibial artery approach was preferred due to the ease of hemostasis. However, the PA approach was selected because both the dorsal and posterior tibial arteries were occluded and could not be selected. The anterolateral PA puncture differs from other puncture methods in that the distance from the body surface to the vessel is longer. Therefore, the height of the puncture site on the body surface and the point at which it reaches the PA are different, and the technique requires familiarity. There are only a few original articles about anterolateral PA puncture and a few case reports on complications. Therefore, we report a case of arteriovenous fistula formation.

The posterior tibial vein joins the fibular vein, which then joins the anterior tibial vein to form the popliteal vein. The vessels below the knee run in a row of arteries and veins. Figure 2e shows the results of post-revascularization angiography with the sheath in place. The guidewire was advanced into the occluded anterior tibial artery. The sheath thought to have been inserted into the PA in the anterolateral PA puncture was inserted into the peroneal artery. Figure 2b shows the vessels involved in the arteriovenous fistula in this case. The artery into which the sheath was inserted was the peroneal artery, and the vein leading to the laterally formed arteriovenous fistula was the anterior tibial vein. The vein leading to the arteriovenous fistula formed on the inside is the fibular vein. This is the first anterolateral PA puncture case performed at our institution, and it is thought that an arteriovenous fistula formed in the peroneal artery and fibular vein during the puncture, and an arteriovenous fistula formed in the anterior tibial vein and fibular artery when the sheath was inserted. The arteriovenous fistula of the peroneal artery and fibular vein was formed with a puncture needle, and the fistula was small; the arteriovenous fistula of the anterior tibial vein and fibular artery was formed with a 4.0 Fr sheath, so the fistula was slightly larger. Therefore, the shunt blood flow is more directed into the anterior tibial vein formed by the insertion of the sheath. The first additional balloon hemostasis eliminated the arteriovenous shunt of the peroneal artery and fibular vein first, and the second balloon hemostasis almost eliminated the shunt blood flow to the anterior tibial vein.

In this case, the sheath was inserted with a small diameter of 4.0 Fr, allowing the patient to be rescued with balloon hemostasis. There are two reasons for the formation of an arteriovenous fistula. First, we were not familiar with the puncture technique, and repeated punctures increased the risk of arteriovenous fistula. This can be overcome by repeated use of the technique to become familiar with the needle entry angle and the distance from the body surface to the PA. The second problem was that the anterior tibial artery was occluded at the entry point, making it impossible to identify the bifurcation between the fibular and anterior tibial arteries during the puncture, resulting in a peripheral puncture. The more distal the needle is, the more veins run parallel to the artery on either side, increasing the risk of an arteriovenous fistula. If the needle can be inserted to reach the PA at the level of the fibular head, the risk of arteriovenous fistula can be reduced. As described above, it is important to recognize that the distance from the body surface to the PA is long in the anterolateral PA puncture and to reach the PA at a high position. By aiming to reach the PA at the height of the fibular head, the risk of a complex arteriovenous fistula involving at least three vessels can be reduced.

## Conclusions

An arteriovenous fistula formed by anterolateral PA puncture and insertion of a 4.0 Fr sheath was successfully bailed out by balloon hemostasis. The anterolateral PA puncture technique requires some practice. However, by recognizing that the distance from the body surface to the PA is 4-5 cm and aiming to reach the PA at the level of the fibular head, the risk of arteriovenous fistula formation can be reduced, enabling a safe distal artery approach.
